# Can we trust naked eye assessments of the capillary refill test in children? An experimental study

**DOI:** 10.1186/s12873-025-01204-0

**Published:** 2025-03-28

**Authors:** Frida Meyer, Jonatan Stahre, Joakim Henricson, Daniel B. Wilhelms

**Affiliations:** 1 Department of Emergency Medicine in Linköping, Department of Biomedical and Clinical Sciences, Linköping University, Linköping, 581 85 Sweden; 2 Crown Princess Victoria’s Child and Youth Hospital, Department of Biomedical and Clinical Sciences, Linköping University, Linköping, Sweden

**Keywords:** Capillary refill, Emergency care, Pediatrics, Microcirculation

## Abstract

**Background:**

The capillary refill test is widely used in pediatric emergency medicine and critical care although its validity and reliability are debated. Naked eye estimation is the recommended method for capillary refill time (CR time) assessment. The goal of this study was to compare naked eye estimations of the CR time in pediatric patients to quantified capillary refill time (qCR time) using polarized reflectance imaging as an objective reference, and to investigate interobserver and intra-observer consistency of naked eye assessments of CR time.

**Method:**

A film sequence comprising videos of capillary refill tests from 15 emergency pediatric patients was shown under standardized conditions to 62 observers (pediatricians, nurses, assistant nurses, and medical secretaries). The observers’ estimations of CR time in seconds and in descriptive categorizations were compared to objectively derived qCR time. Three tests were shown twice without the observers’ knowledge.

**Results:**

There was poor interobserver agreement in all professions, with limits of agreement ranging from 1.17 s (assistant nurses) to 2.00 s (secretaries). Intra-observer agreement for estimations of both time and descriptive categorizations was limited. The correlation between naked eye assessments and qCR time was weak.

**Conclusion:**

This study shows that naked eye assessment of CR time in children is highly subjective with poor reproducibility in pediatric nurses and pediatricians, as well as in comparison to a quantitative method. Based on the lack of both inter- and intra-observer consistency in the assessments, these findings suggest that CR time assessed by naked eye should be questioned as a routine test in pediatric emergencies.

## Introduction

The capillary refill test (CR test) originates from experiences in assessing various degrees of shock in wounded soldiers during World War II and is now globally implemented as part of several frameworks for assessment of critically ill and injured [1]. The test is widely used in emergency pediatric patients to assess circulatory status. Although it was originally designed for evaluating hemorrhagic shock, it is now applied to assess circulatory compromise regardless of its cause. It is recommended in the Pediatric Advanced Life Support Course and has also been recommended by the World Health Organization in its guidelines for the care of critically ill children [2].

Recently, a novel screening tool for sepsis in pediatric patients, qPS4, has been suggested, which includes the CR test [3]. A plausible reason for the popularity of the CR test among pediatric health care providers is its non-invasive and pain-free nature, and ease of performance, regardless of available equipment or environmental conditions.

Although widespread, there is no broadly accepted standardization on how the test should be performed [4] and the capillary refill test has not been validated against a criterion standard, which makes studies of the CR test difficult. In clinical practice, the CR test is commonly performed by the examiner pressing his or her index finger against the skin on the sternum, distal phalanx or the nailbed of the finger or toe of the patient for five or ten seconds. After releasing the blanching pressure, the examiner assesses with the naked eye the time in seconds it takes for the skin color to return to the same color as before the pressure was applied. Commonly, more than two seconds is regarded as prolonged CR time and a sign of circulatory compromise [5]. The test, as commonly performed, could be regarded as highly subjective. The use of a chronometer is uncommon. An objective method for reference is hence helpful in studies of the CR test. In the present study we used polarized reflectance imaging, as suggested by John et al. [6] to obtain objective quantified CR (qCR) time curves. The method generates a value that correlates to the concentration of red blood cells and thus the erythema intensity within the measurement area. At video mode, the system captures the refilling process with a high temporal and spatial resolution, which allow for exact calculation of the refill time.

There is consensus in the pediatric community that a prolonged CR time is associated with an increased risk of adverse outcomes. These claims have been supported by some studies, both in pediatric and adult patients [7–9]. Other studies question the use of the CR test due to its poor interrater reproducibility [10–14]. However, few studies, if any, have investigated this relationship in detail. Despite the controversy on the validity of the CR test, it is still widely used in clinical practice. Recommendations to use the test in screening tools and checklists for acutely ill children reinforce the assumption among clinical staff that the test is accurate and valid.

In recent years, the view of the CR test has changed, from being seen primarily as a proxy to macrocirculatory function, to more often described as an indicator of how well the microcirculation functions. The CR test has recently been recommended as a test of microcirculatory function [15]. The need for research regarding macro- and microcirculatory changes in pediatric septic shock is currently discussed, where the integration of measurements of macro-, microcirculation and tissue metabolism is in focus [15]. Prolonged CR time has been associated with microcirculatory changes visible with video microscopy in pediatric sepsis patients admitted to a pediatric intensive care unit [16], but there is currently no standardized method used in clinical practice that can quantify microcirculatory changes.

## Methods and materials

The primary objective of this study was to compare the naked eye CR assessments in seconds made by pediatricians, nurses, assistant nurses and medical secretaries in a pediatric emergency department setting with the machine-derived qCR times in seconds. The second objective was to compare the naked eye categorical CR assessments made by each observer with qCR time and common guidelines. The objective was also to investigate intra-observer repeatability in naked eye CR time assessments for each profession, and to investigate any differences in naked eye CR time estimates in seconds among the various professions.

The tertiary objective was to investigate within group variability for naked eye CR time and categorical CR assessments made by each profession.

### Study design

This was an experimental study in pediatricians, nurses, assistant nurses, and medical secretaries. The video films of tests generated during the qCRtest were used to generate a film sequence presenting tests for assessment by the observers. Three of the fifteen tests were presented twice as a test of repeatability.

The study was approved by the regional ethical review board of Linköping, Sweden, approval number 2018/134 − 31. All participating health care providers and the legal guardians of the pediatric patients from whom the CR test videos were obtained gave written and oral consent prior to participation.

### Study setting

The study was executed in the pediatric emergency department of Linköping University Hospital, Sweden. Data was collected in November and December 2019. Fifteen patients with various acute conditions of different severity seeking the pediatric emergency department were included. The age of the patients ranged from post neonatal period to 5 years old. CR tests were performed on the sternum of the patients and video filmed by a member of the research group.

From each CR test video, an objectively quantified CR time (qCR time) was derived. The CR test videos (patient cases) were then compiled in a randomly organized sequence. Three CR test videos were used twice, and the film sequence thus included 18 cases.

A convenience sample of 15 pediatricians, 16 nurses, 16 assistant nurses and 15 medical secretaries, all working at the Pediatric Clinic and the Emergency Department at Linköping University hospital, were recruited as observers. The medical secretaries represented non-medical professionals without clinical experience. The median (range) work experience in years for the pediatricians was 10 (0.25 to 30), nurses 10 (1 to 30) and assistant nurses 5 (1 to 41).

### Study protocol

Capillary refill tests were performed on the patients’ sternum by application of a blanching pressure by the investigators’ (authors FM or JS) index fingers. The procedure was filmed using a 3D-printed tube attached to the camera that was in contact with the patients’ skin to guarantee consistent light conditions and distance (5 cm) between the lens and the skin. Refill tests were performed using a 5 s baseline, 5 s of pressure and 10 s for refill, with the camera recording continuously during these 20 s. The skin on the sternum was chosen as it is less susceptible to ambient room temperature than the skin on the fingers and as it is the recommended anatomical site for performing the test in clinical practice at the hospital where the study was performed.

The film sequence of 18 CR test cases was shown once to every observer. The observers were asked to estimate the CR times in seconds and descriptively using one of the three categories “normal”, “slow,” or “definitely sluggish”. These categories were used in Beecher’s original work [1] without any further descriptions or definitions. “Definitely sluggish” was defined as slower than “slow” in this study if the observers asked for the definitions. The observers were presumed to have basic knowledge of the conduct and interpretation of the CR test, except for the secretaries who were given short general information about the test. Observers were not given any instruction on whether to use whole or fractions of seconds for their time estimates.

The tissue viability imaging system, TiVi (WheelsBridge AB, Linköping, Sweden), used in this study as an objective reference method utilizes a standard digital camera (Canon EOS M100) in HD video mode (1280 × 720 pixels) at 50 frames per seconds equipped with a Canon EF-M 28 mm f/3,5 Macro IS STM objective with a built-in light source (white LEDs). The imaging software uses the wavelength dependent differences in absorption in the wavelength region 500 to 700 nanometers between red blood cells and surrounding tissue to generate a tissue viability value (TiVi-value) for every pixel. The TiVi-value correlates linearly to the concentration of red blood cells within the measurement volume [17].

**Fig. 1:**
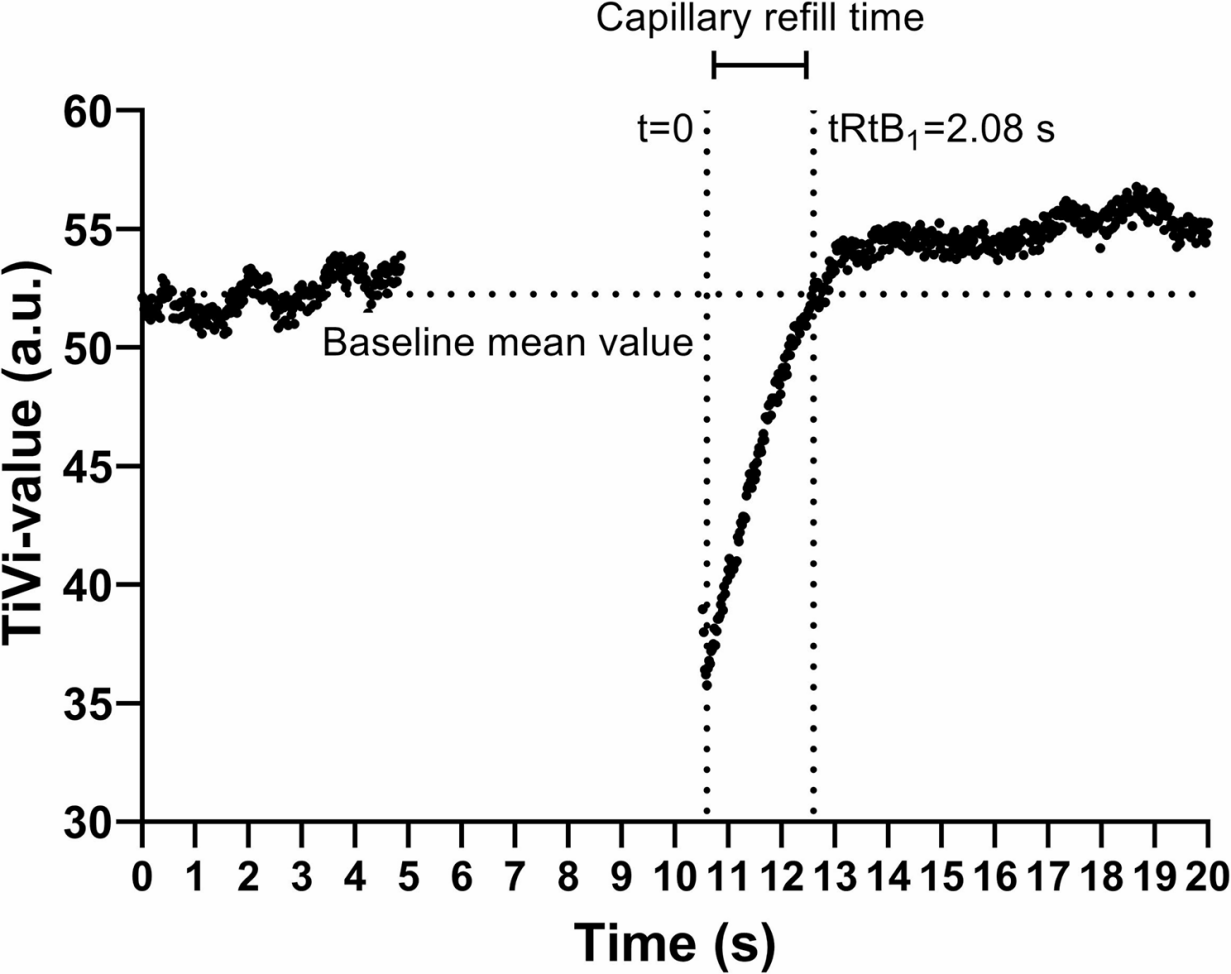
An example of a machine derived quantified capillary refill CR (qCR) time curve. Time to return to baseline (tRtB1) is the time in seconds needed for the erythema intensity (as measured using polarized reflectance imaging) to return to baseline intensity (dashed horizontal line) after the release of a blanching pressure

Analysis began by separating each video into single frames. A region of interest (ROI) was applied in every single image within the blanched skin area, generating a mean TiVi-value for each ROI. Mean TiVi-values were used to generate graphs showing the dynamics of the refill process (Fig. 1). The qCR time was acquired by calculating the mean TiVi-value of the first 250 ROIs (Baseline mean value – dashed horizontal line in Fig. 1) and refill time was defined as the time for the blanched area to have a TiVi-value equal to or greater than the baseline value. This time was denoted time to return to baseline 1, (tRtB1) [6].

A qCR time up to 2.0 s was classified as “normal”, longer than 2.0 s as “slow” and a time longer than 3.0 s as “definitely sluggish”.

### Data analysis

The outcomes were: inter-observer variability, indicated by Limits of Agreement (LoA) for the quantitative CR estimates for each profession; Fleiss’ Kappa for interobserver agreement on categorical judgement between observers within each profession; correlation coefficient (R^2^) between naked eye CR time estimates for each profession and qCR times; percental agreement for the categorical naked eye estimations of CR time and machine-derived categorical CR measurements; percental agreement for intra-observer repeatability.

Statistical tests were performed using GraphPad Prism version 8.4.3 (686) for Windows 64-bit, GraphPad Software, San Diego, California USA, www.graphpad.com and Microsoft Corporation, 2018. Microsoft Excel, Available at: https://office.microsoft.com/excel.

**Fig. 2:**
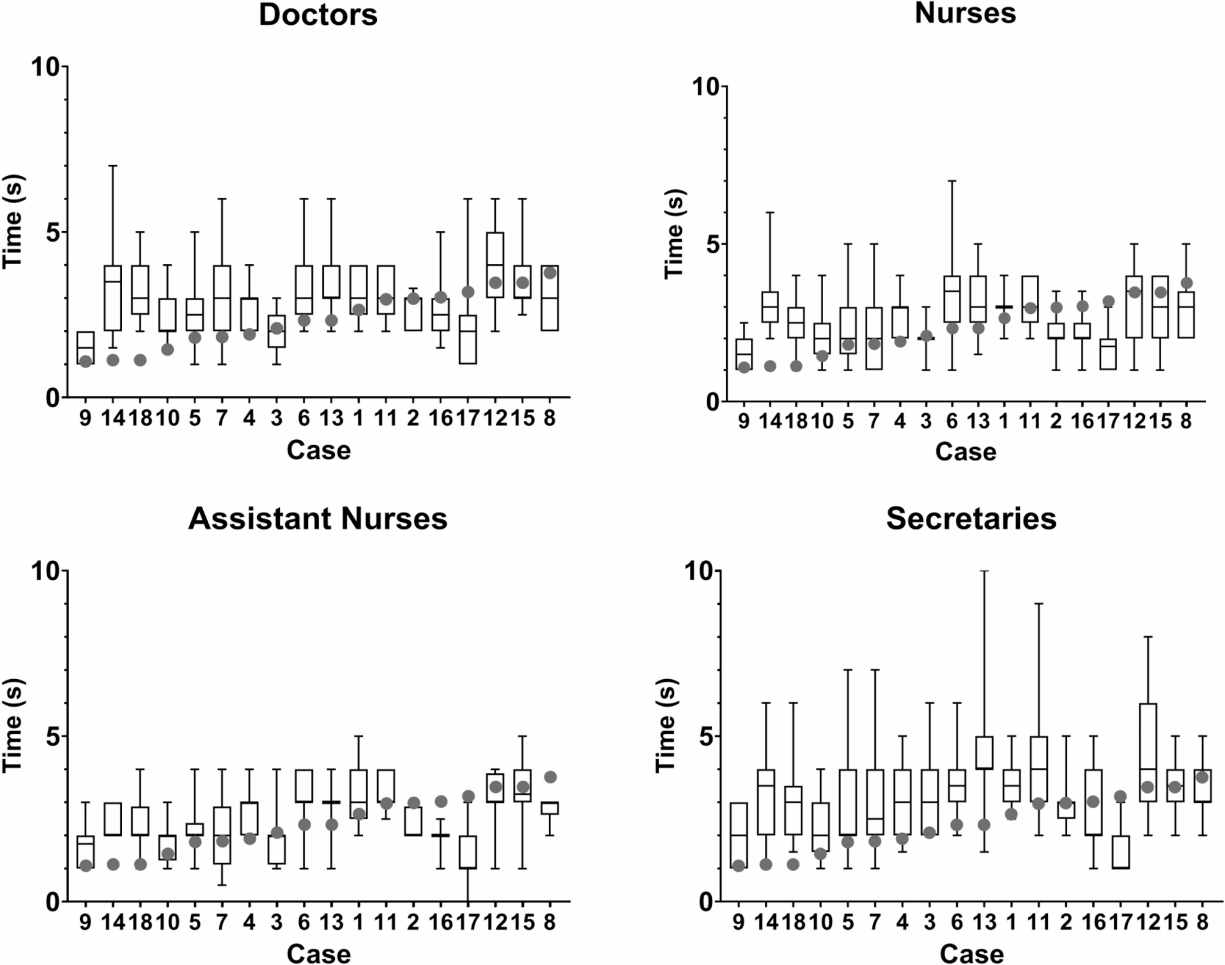
Boxplots of the naked eye estimation of CR time shown in seconds, including median (horizontal line), the 25th to 75th percentile (box) and range (error bars) of the values. Quantified capillary refill time, qCR time, in seconds (tRtB1, grey dot) is superimposed for each case for comparison. The cases with the shortest tRtB1 values (qCR times) are plotted to the left and the longest to the right on the X-axis. The number of each case indicates the order in which they appeared to the observers in the sequence. Three video films were shown two times without the observers’ knowledge. Cases 14 and 18 are identical, as are cases 6 and 13, and cases 12 and 15.

The within-group variability for the quantitative naked eye assessment of CR time was initially shown graphically by constructing boxplots of the time estimates for each profession and case (Fig. 2). The qCR times for each case were superimposed (grey circles, Fig. 2) for comparison.

Fleiss’ kappa was run to determine agreement on categorical judgement within each profession on whether the capillary refill times were either “normal”, “slow”, or “definitely sluggish”, based on a video clip showing each refill test performed on 15 unique pediatric patients. Fifteen pediatricians and administrative staff members as well as 16 nurses and assistant nurses were chosen at random from a group of medical staff members at the pediatric emergency department in Linköping to rate each patient. Each observer rated the video clip separately to not influence the decision of the other observers. When assessing the refill tests, each observer could select from only one of the three categories: “normal”, “slow” or “definitely sluggish”.

**Fig. 3:**
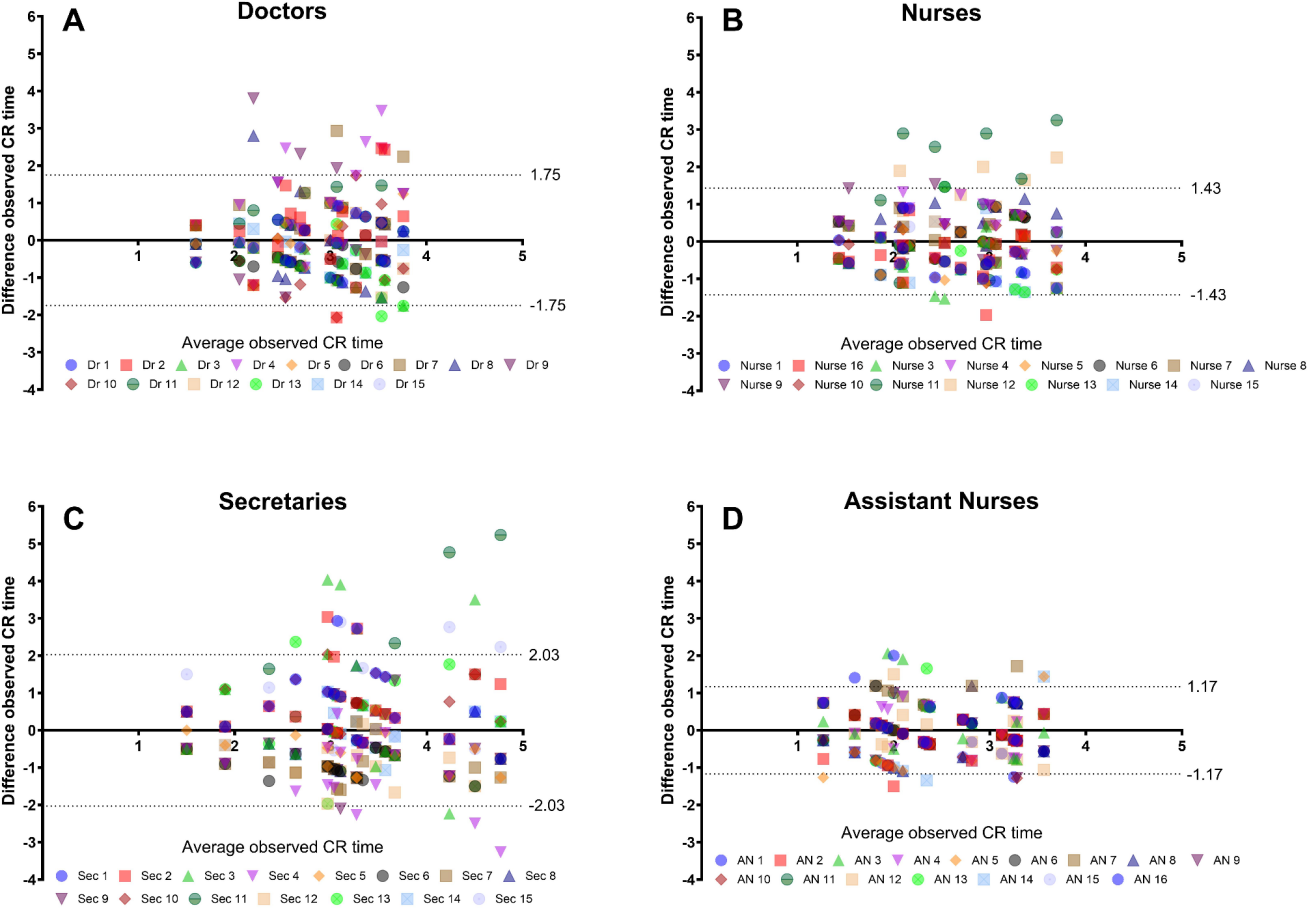
(**A**-**D**). Multiple observer Bland-Altman plot for interobserver variability of naked eye assessed CR time in seconds shown by profession, with each figure/form in the graphs representing one observer. The 95% limits of agreement with the mean of all observers are shown as dashed lines. The limits of agreement are broad for all professions, regarding the normal time limit of the CR test

The inter-observer variability within each profession was investigated further by construction of multiple observer Bland-Altman plots (Fig. 3). Regular One-way ANOVA with Tukey’s post hoc test was used to investigate differences in naked eye quantitative CR time estimates (in seconds) between the various professions.

## Results

We recruited 62 observers, all fair skinned, that generated in total 98% complete naked eye CR time in seconds and categorical estimations. One observer failed to assess any of the cases in seconds and was excluded from the study. One observer failed to assess one case in seconds. Two observers gave no categorical assessments to one and two cases, respectively.

Most naked eye estimations were made in whole seconds, rather than half seconds. The use of whole seconds was more common among medical secretaries and assistant nurses.

### Comparison between quantitative naked eye assessment and qCR

Capillary refill (CR) time assessments made by the naked eye for each profession and case were represented as boxplots (Fig. 2). Quantified capillary refill times in seconds (tRtB1), depicted as dark grey circles, were superimposed for each case to facilitate comparison. Cases were arranged in ascending order along the x-axis, with the shortest tRtB1 values positioned on the left and the longest on the right.

Correlation analysis showed there were weak, positive correlations between quantitative CR time estimates for the different professions, and machine derived qCR times, doctors; Pearson’s r (270) = 0.14, *p* < .002; nurses; r (269) = 0.19, *p* < .001; assistant nurses; r (287) = 0.29, *p* < .001; secretaries; r (269) = 0.15, *p* = .013.

### Categorical assessments

The categorical estimations were compared to the qCR generated categorical classifications of the cases. The agreement between the observers’ categorical estimations and qCR classifications was low; 41% in doctors 44% in nurses 42% in medical secretaries and 44% in assistant nurses.

### Intra-observer repeatability

**Fig. 4:**
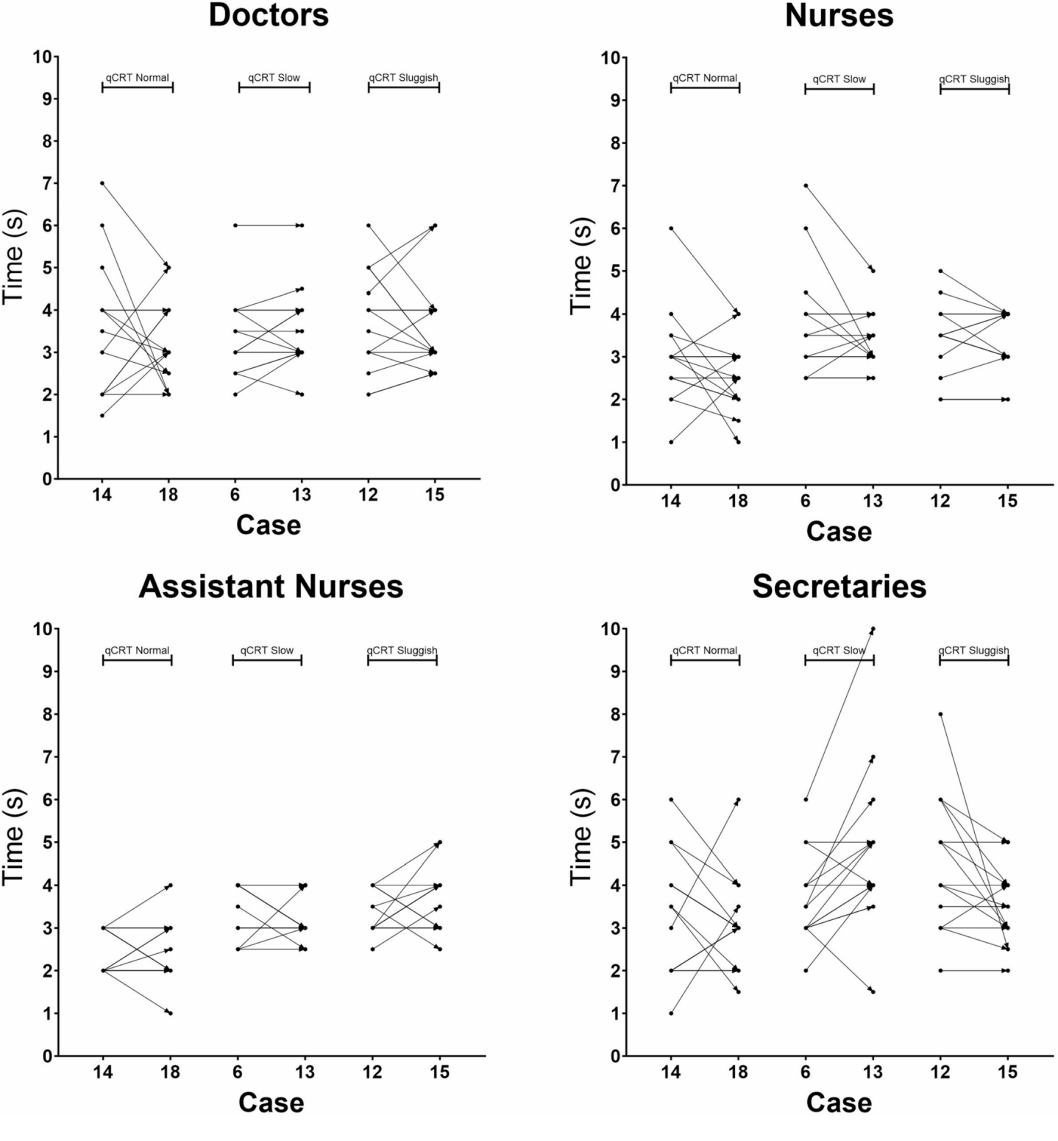
Estimations of naked eye CR time in seconds for the three films that were shown twice without the observers’ knowledge. Cases 14 and 18 had a qCR time of 1.12 s, 6 and 13 a qCR time of 2.32 s, and 12 and 15 a qCR time of 3.46 s. Thus, according to the most used criteria for CR time, these CR times were classified as “Normal”, “Slow” and “definitely sluggish”

To investigate intra-observer repeatability, three cases were shown twice without the observers’ knowledge. For the naked eye estimation in seconds, consistency between the first and second estimate was low for all professions (Fig. 4). Intra-observer consistency shown as percentage of answers having identical quantitative estimations in seconds after the first and second time watching the three repeated films is shown in Table 1. Intra-observer consistency shown as percentage of answers having identical categorical estimations (“normal,” “slow”, “definitively sluggish”) after the first and second time watching the three repeated films is shown in Table 2.

**Table 1:** Intra-observer consistency is shown as percentage of answers having identical quantitative estimations of CR time between the first and second time watching the three repeated cases. The number of individuals in each professional group is shown in brackets

Profession	Cases 14 and 18	Cases 6 and 13	Cases 12 and 15
Doctor (15)	20%	27%	13%
Nurse (15)	27%	53%	26%
Assistant Nurse (16)	44%	69%	38%
Medical Secretary (15)	13%	20%	33%

**Table 2:** Intra-observer consistency is shown as percentage of answers having identical categorical estimations (normal, slow and definitively sluggish) of CR time between the first and second time watching the three repeated cases. The number of individuals in each professional group is shown in brackets

Profession	Cases 14 and 18	Cases 6 and 13	Cases 12 and 15
Doctor (15)	33%	67%	53%
Nurse (15)	53%	73%	60%
Assistant Nurse (16)	79%*	56%	75%
Medical Secretary (15)	63%	38%	31%

*Two assistant nurses assessed the case only once

### Difference in naked eye quantitative CR time estimates between professions

A significant difference in naked eye CR time estimates between groups (professions) could be found only for cases 2, 3, 11, 12, 14 and 18 (one-way ANOVA). In cases 2 and 12 post hoc tests (Tukey) revealed a significant difference for nurses, assistant nurses, and medical secretaries. In cases 3 and 14 significant differences were found between all other professions and medical secretaries. In cases 11 and 18 significant differences were found between nurses and medical secretaries, and doctors and medical secretaries, respectively.

### Within-group variability for naked eye assessments in seconds

The boxplots for time estimates by each profession and case indicated a considerable within-group variability for the quantitative naked eye assessment of CR time (Fig. 2).

As seen in the multiple-observer Bland-Altman plots (Fig. 4), the limits of agreement were wide for all professions (doctors 1.75 s, nurses 1.43 s, medical secretaries 2.03 s and assistant nurses 1.17 s) regarding the most recommended cutoff (2 s) for a normal CR test.

### Within-group variability for categorical naked eye assessments

Fleiss’ Kappa showed that there was poor agreement between the observers’ judgements for the categorical naked eye assessments (normal, slow, definitely sluggish) for all professions; doctors: κ = 0.167 (95% CI, 0.131 to 0.203), *p* < .001; nurses; κ = 0.163 (95% CI, 0.127 to 0.199), *p* < .001; assistant nurses: κ = 0.186 (95% CI, 0.147 to 0.224), *p* < .001; secretaries; κ = 0.111 (95% CI, 0.73 to 0.148), *p* < .001.

## Discussion

This study shows that even pediatric medical staff with several years of work experience struggle to consistently estimate CR time, and that naked eye estimations of CR time show poor agreement. This is in line with findings from studies of CR time estimations made by experienced medical staff providing emergency medical care for adult patients [10, 13, 14, 18]. Our results strongly suggest that the use of naked eye CR test in the assessment of pediatric patients should be questioned. It is also notable that the observations of medical secretaries only show significant differences to other professions in case 11 and 18. If medical knowledge or clinical experience would affect the accuracy of estimations of CR time, the medical secretaries as a professional group would differ more from the medical professional groups in this study.

The difficulties in estimating CR time for health care staff in a reproducible way may have several causes. To estimate CR time, two tasks must be performed simultaneously – counting time and processing visual information (the change of color in the skin). There is ample evidence supporting that doing a task while estimating time makes time estimation more difficult since the cognitive capacity will be split between two demanding tasks [19, 20]. Hence, using a stopwatch for estimating time in CR tests could make the estimation less susceptible to error from sharing attention between two tasks, but the use of a stopwatch is not supported by guidelines nor part of medical practice. In the study by Hernandez et al., a chronometer was used when assessing the time for return to normal skin color, and the study suggested lower mortality and less organ dysfunction in patients where CR time was measured [21].

The examiner will probably evaluate the skin prior to the CR test and look for pallor or redness. It is unknown to what degree humans can remember the exact nuance of the skin as it was before the CR test. However, contrast sensitivity is important in human vision, but the process of contrast information is not fully explored and is to some extent related to temporal changes of contrast [22].

Except for visual information, the examiner is subject to other sensory inputs when performing a CR test. The examiner touches the patient’s skin and feels the temperature of the skin, as well as its turgor. All these sensory inputs are incorporated with the history of the patient and other findings, into the overall assessment of the patient where biases might play a role [23]. This could influence the interpretation of the CR test, e.g., if the patient has an alarming history or other vital signs are indicative of a severe condition, the CR time could be estimated longer than it is. This is not tested for in studies but could explain why some studies find CR tests suitable for assessing pediatric patients.

It is unclear how the CR test has become so prevalent in healthcare, and especially in pediatric care, since the validity of the test is unknown, and its usefulness has repeatedly been questioned. One reason for its popularity among pediatric health care staff could be that the test is quick, noninvasive, and simple, making it uncomplicated to perform even if the child is upset or unwilling to be examined. Another reason could be the debate about blood pressure measurements in children to evaluate the degree of shock as low blood pressure can develop very shortly before circulatory arrest, and hence other circulatory markers, such as CR time, has been of particular interest [24].

In this study, we are unable to determine the precise reasons why even experienced healthcare professionals have difficulty assessing CR time. The test, at least as it is currently carried out, is fraught with such uncertainty in naked eye assessments that it is not even possible to take a credible position on its potential link to abnormal physiological processes. Until the possible link between an abnormal capillary refill reaction to relevant pathophysiological processes has been determined, the test should be used with due caution to these variables.

A limitation of this study was the anatomical site used for performing the CR test. The sternum is the preferred anatomical site for testing CR time in some parts of the world and the recommended site at the hospital where the study took place, but most research on the CR test has focused on the distal phalanx of the finger. Normal CR times vary depending on which anatomical site is used for CR test.CR time in children for CR test on the sternum is regarded normal up to 4 s in some studies [25], but it is unclear if clinical staff is aware of the differences in normal CR time in different anatomical sites. Another limitation of this study was the choice of ROIs in the videos of the CR tests. The ROI was manually selected from the area that appeared to be most blanched, but both the placing and the size of the ROI could affect the qCRtime, which was used as standard for reference. Yet another limitation was the skin pigmentation of the pediatric patients included. All patients had fair skin which is thought to be easier to assess than dark skin regarding capillary refill. Most of the research on the CR test only includes fair skin patients whilst most of the world’s population have darker pigmentation of the skin. This is an aspect of equality that needs to be addressed as large groups of patients are at risk of being wrongly assessed if knowledge on the validity of the test in dark skin is not investigated.

## Conclusion

In this study, we show that naked eye assessments of CR time in children showed low agreement with an objective quantitative skin physiological method. Naked eye assessments of CR time in children are characterized by high inter-rater variability, and poor reproducibility in pediatric staff. These findings suggest that the CR test as presently used in pediatric clinical practice should be interpreted with caution.

Future studies of the technique’s usefulness should include qCR assessment in order to establish an objective reference value in interpretation in any validating comparison with clinical findings and outcome.

## Data Availability

The datasets generated and/or analyzed during the current study are not publicly available due to integrity of study subjects but are available from the corresponding author. On reasonable request data will be available from the author.

## Declarations

### Prior presentation of study data

Parts of the results have previously been presented as an abstract and a poster at the at the SPIE Photonics West Conference in 2023.

## Abbreviations

CR time: Capillary refill time
CR test: Capillary refill test, tRtB1 – time to return to baseline 1
TiVi: Tissue viability imaging
TiVi-value: Tissue viability imaging value
qCR: Quantified capillary refill
